# Pre-travel health awareness and perceptions of voluntary airport PCR testing during COVID-19: A cross-sectional study in Okinawa, Japan

**DOI:** 10.1016/j.ijregi.2025.100817

**Published:** 2025-11-30

**Authors:** Masahiro Kozuka, Yusuke Shimakawa, Gerardo Chowell, Tetsuharu Nagamoto, Ryota Matsuyama, Ryosuke Omori, Yining S. Xu, Yoshiro Shimoji, Kaoru Ogawa, Yoshihiro Takayama, Kenji Mizumoto

**Affiliations:** 1 Graduate School of Advanced Integrated Studies in Human Survivability, Kyoto University, Kyoto, Japan; 2 Okinawa Prefecture Commission for Epidemiological and Statistical Analysis, Okinawa, Japan; 3 Unité d'Epidémiologie des Maladies Emergentes, Institut Pasteur, Paris, France; 4 Pasteur International Unit at Kumamoto University, National Center for Global Health and Medicine, Tokyo, Japan; 5 International Research Center for Medical Sciences, Kumamoto University, Kumamoto, Japan; 6 School of Public Health, Georgia State University, Atlanta, USA; 7 Graduate School of Informatics, Kyoto University, Kyoto, Japan; 8 Rakuno Gakuen University, Hokkaido, Japan; 9 Division of Bioinformatics, International Institute for Zoonosis Control, Hokkaido University, Hokkaido, Japan; 10 Japan Okinawa Convention & Visitors Bureau, Okinawa, Japan; 11 Okinawa Prefectural Chubu Hospital, Okinawa, Japan; 12 Graduate School of Biomedical Sciences, Nagasaki University, Nagasaki, Japan

**Keywords:** Fever screening, PCR testing, COVID-19, Airport, Survey

## Abstract

**Objectives:**

During the COVID-19 pandemic, airport-based measures, such as fever screening and polymerase chain reaction (PCR) testing were implemented in Japan. Okinawa Prefecture introduced a voluntary airport PCR testing program for domestic travelers at Naha Airport (OKA). Their indirect behavioral effects on travelers remain underexplored. This study aims to describe self-reported pre-travel health awareness among participants in this program.

**Methods:**

In February-March 2021, we conducted a cross-sectional questionnaire survey among Naha Airport PCR test Project (NAPP) participants (n = 4545; March subset n = 1859). The survey assessed demographics, travel purpose, awareness of airport screening (fever screening and PCR testing), pre-travel health awareness, COVID-19 history, and symptoms. Logistic regression evaluated factors associated with self-reported symptoms in the March subset.

**Results:**

Among respondents aware of fever screening and PCR testing, 94.1% and 96.4%, respectively, reported increased attention to their physical condition before travel. Overall, 3.9% reported symptoms, mainly mild respiratory complaints. The proportion symptomatic varied by reason for testing; workplace-mandated testers reported fewer symptoms than family-motivated testers (adjusted odds ratio 0.36, 95% confidence interval 0.15-0.78).

**Conclusions:**

Awareness of voluntary airport screening measures was associated with greater self-reported pre-travel health awareness among voluntary testers. These findings may inform context-specific behavioral strategies aimed at promoting health-conscious travel during infectious disease outbreaks.

## Highlights

• Voluntary airport polymerase chain reaction testing was offered to domestic travelers in Okinawa.
• Over 95% of travelers aware of screening reported increased pre-travel health awareness.
• Self-reported symptom proportion was 3.9%, mostly mild respiratory complaints.
• Workplace-mandated testers reported fewer symptoms than family-motivated testers.
• Voluntary screening may be a behavioral nudge in future infectious disease control.

## Introduction

During global health emergencies, such as the COVID-19 pandemic, many countries have implemented airport-based screening measures—including thermographic fever screening (hereafter, ‘fever screening’) and polymerase chain reaction (PCR) testing—to mitigate the introduction of infectious diseases via air travel [[1], [2], [3], [4]]. These measures, often combined with mandatory negative test certificates, documentation of recovery, or quarantine upon departure or arrival, aim to identify infected travelers and physically isolate those with fever or positive test results. Although their direct effectiveness is limited, these visible screening measures may exert important indirect behavioral effects on travelers.

Regarding the direct effect of fever screening as a border control measure at arrival airports, observational and modeling studies from the 2009 H1N1 pandemic and COVID-19 suggest that it can detect some infected passengers, but its overall effectiveness in preventing their importation is limited [[5], [6], [7], [8], [9], [10], [11]]. Beyond this limited direct effect, it has been hypothesized that the presence of fever screening at airports may promote behavioral change in travelers and strengthen self-management of their physical condition and health awareness before travel; however, little is known about the indirect effects on passengers’ pre-travel health awareness.

Unlike travel to foreign countries, reverse transcription-PCR testing before boarding and after arrival was not required for domestic flights in Japan, and neither negative test certificates nor vaccine certificates were mandated [12,13]. In addition to fever screening at domestic airports, passengers were required to wear masks onboard and voluntarily refrain from boarding if they felt ill. In this context, Okinawa Prefecture, whose main gateway is by air and is a major tourist destination, launched an out-of-pocket PCR testing program at OKA—the Naha Airport PCR test Project (NAPP)—in February 2021 [14].

This study aims to describe self-reported pre-travel health awareness and related factors among domestic travelers, and to examine how awareness of airport-based health measures and reasons for testing are associated with pre-travel health awareness and self-reported symptoms, using a questionnaire survey of NAPP participants. By characterizing these behavioral patterns among voluntary testers, this study provides evidence to inform traveler-centered, context-specific strategies for future infectious disease preparedness and airport-based health interventions.

## Material and methods

### Naha airport PCR test project

PCR tests for COVID-19 operated by Okinawa Prefecture were mainly for arriving passengers from epidemic regions within Japan on domestic flights from February 3, 2021, to March 31, 2021, who could not take PCR tests before travelling due to unavoidable circumstances [14]. Registrations were accepted online, and reservations could be made 2 days in advance. The PCR test cost 5000 JPY(Japanese yen) for Okinawa residents and 7000 JPY for non-residents (approximately 32 and 45 US$, respectively). Participants self-collected saliva on site using collection kits and submitted specimens immediately; results were generally returned the same day for morning submissions and the next day for afternoon submissions.

### Study setting

In response to the rapid spread of COVID-19 since the beginning of 2021, the Okinawa Prefectural Government issued an Okinawa Prefecture Emergency Declaration on January 20, 2021, requesting residents to refrain from going out and to avoid non-essential travel—both between Okinawa’s remote islands and between Okinawa and prefectures under a national or prefectural state of emergency [15]. The prefecture also recommended pre-travel PCR testing for passengers arriving from those affected regions [16]. As of January 18, 2021, one-third of prefectures, including large metropolitan prefectures such as Tokyo, Chiba, Kanagawa, and Osaka, were under a state of emergency, indicating that the epidemic was spreading throughout Japan [15].

We conducted a cross-sectional questionnaire survey from February to March 2021 among domestic travelers arriving at OKA, who voluntarily participated in the NAPP implemented by the Okinawa Prefectural Government. The questionnaire collected information on place of residence, age group, purpose of travel, and reason for testing to characterize PCR applicants, and previous awareness of fever screening and PCR testing for febrile passengers at the airport to gauge how widely these measures had been communicated. An additional questionnaire was administered in March 2021, covering passenger backgrounds related to COVID-19 infection, protection measures, and physical conditions with subjective symptoms.

In this study, “fever screening” denotes thermographic screening of passengers at the airport, with PCR testing offered to those identified as febrile. The study period captured the earliest phase of the voluntary airport-based PCR initiative, providing a consistent operational environment before major changes in national testing policy.

### Data sources

In this study, “pre-travel health awareness” was defined as an increased level of self-monitoring of physical condition before travel, as self-reported by participants in response to screening-related questions.

The questionnaire comprised eight main items, including questions about personal background (e.g., age, gender, residence), purpose of travel, reason for testing, previous awareness of fever screening and PCR testing for febrile passengers, and pre-travel health awareness related to these screenings.

An additional questionnaire administered exclusively in March 2021 focused on passenger backgrounds related to COVID-19 infection, protection measures, and physical conditions with subjective symptoms, travel history, and source of information about NAPP.

The number of newly confirmed cases of COVID-19 in Okinawa Prefecture and all prefectures in Japan was collected from a database operated by the Ministry of Health, Labour, and Welfare of Japan [17].

The questionnaire was not formally validated, and response rates per item varied; missing data were excluded using listwise deletion, and self-selection bias is acknowledged as a limitation. The questionnaire dataset provided to the research team was de-identified and not linkable to laboratory results managed separately by contractors; therefore, respondent-level PCR positivity cannot be calculated. All analyses were conducted on anonymized data approved for research use by the Okinawa Prefecture Government.

### Statistical analysis

Confidence intervals were obtained using the exact binomial test. The Wilcoxon rank sum test with continuity correction was used to analyze the age distribution across travel purposes. A two-sample test for equality of proportions without continuity correction was used for binary (two-choice) questions. Two-sided *P* <0.05 was considered statistically significant; Bonferroni adjustment was applied where multiple comparisons were performed. For contextual incidence, daily cases per 100,000 were computed from the Ministry of Health, Labour and Welfare (MHLW) dataset as 7-day moving averages using October 1, 2021, population denominators for Okinawa and for Japan overall; detailed data processing steps are described in the Supplement.

### Statistical analysis – March 2021 sub-analysis (symptoms and correlates)

For the March 2021 OKA subset, we modeled symptomatic status (“with” vs “without” symptoms) as a binary outcome. We first performed univariate logistic regression analyses, treating each explanatory variable (age group, gender, vaccination history, previous COVID-19 infection, residence, and reason for testing) in turn. We then fitted a multivariable logistic regression model with symptomatic status as the outcome and reason for testing as the main exposure, adjusting for age group, gender, vaccination history, previous COVID-19 infection, and residence. We report adjusted odds ratios (aORs) from the multivariable logistic model as the primary effect estimates; as a sensitivity analysis, we additionally fitted a multivariable Poisson regression model with robust variance to obtain adjusted risk ratios (aRRs); full model specifications and results are presented in Supplementary Tables S3-S4. The purpose of travel was examined descriptively (Table 4) but was not included in the regression models because of its strong alignment with the reason for testing (e.g., visiting friends/relatives vs family request, business travel vs workplace mandates) and the limited number of symptomatic events, which would have led to unstable estimates.

All statistical analyses were conducted using R version 4.5.1 (R Foundation for Statistical Computing, Vienna, Austria).

## Results

Among the 5308 passengers who took PCR tests in the NAPP project, a total of 4545 arriving passengers at OKA were included as survey participants for this study. The average age of participants was 38.7 years. The two largest age groups were the 20s (23.2%, 1056/4545) and the 50s (20.3%, 923/4545) (Table 1). Males constituted 54.3% (n = 2469) of participants, and residents of Okinawa comprised 66.0% (n = 2998). Approximately one-third of participants (n = 1547, 34.0%) were from outside Okinawa Prefecture. Among respondents who reported their travel purpose (n = 4386), the most common was visiting friends and relatives (43.5%), followed by business (24.1%) and sightseeing (4.6%). The age distribution was bimodal, with peaks in the 20s and 50s, mainly corresponding to travelers visiting friends/relatives and those traveling for business, respectively (Figure S1). This bimodal pattern reflects distinct traveler profiles and supports the stratified analyses presented below. Among respondents who stated their reason for testing (n = 4199), the largest proportion cited family request (52.1%), followed by workplace mandate (24.0%) and personal concern (17.2%) (Table 1). Reasons for testing closely aligned with travel purpose, illustrating consistent behavioral patterns across subgroups.

**Table 1:** Participant characteristics and travel/testing context among Naha Airport polymerase chain reaction Project participants (n = 4545).

**Panel A. Demographics** (n = 4545)							
		Mean (SD) or n (%)					
Age		38.7	(16.5)				
Age group	Under 10	88	(1.9)				
	10s	518	(11.4)				
	20s	1056	(23.2)				
	30s	640	(14.1)				
	40s	842	(18.5)				
	50s	923	(20.3)				
	60s	379	(8.3)				
	70s	83	(1.8)				
	80s	15	(0.3)				
	Over 90	1	(0.0)				
Gender	Male	2469	(54.3)				
	Female	2074	(45.6)				
	Other	2	(0.0)				
Residence	Okinawa	2998	(66.0)				
	Outside Okinawa	1547	(34.0)				
Date of participation	February 2021	1923	(42.3)				
	March 2021	2622	(57.7)				

**Panel B. Purpose of travel** (n = 4386; response proportion = 96.5%)							

			Residence				*P*-value^a^
	All participants		Okinawa		Outside of Okinawa		
	n	(%)	n	(%)	n	(%)	

Visiting friends and relatives	1906	(43.5)	1242	(43.0)	664	(44.1)	0.504
Business	1056	(24.1)	617	(21.6)	439	(29.2)	< 0.001^b^
Sightseeing	200	(4.6)	95	(3.7)	105	(7.0)	< 0.001^b^
Other	1224	(27.9)	928	(31.7)	296	(19.7)	< 0.001^b^
Total	4386	(100)	2882	(100)	1504	(100)	-
**Panel C. Reason for testing** (n = 4199; response proportion = 92.4%)							
For family	2187	(52.1)	1472	(53.1)	715	(50.0)	0.056
Workplace mandates	1006	(24.0)	597	(21.6)	409	(28.6)	<0.001^b^
Personal concern	724	(17.2)	545	(19.7)	179	(12.5)	<0.001^b^
Other	282	(6.7)	156	(5.6)	126	(8.8)	<0.001^b^
Total	4199	(100)	2770	(100)	1429	(100)	-

a Bonferroni-adjusted *P*-values from a two-sample test for equality of proportions (no continuity correction); *P* <0.0125 regarded as statistically significant for this table.

b *P* <0.001.

To describe self-reported health awareness in relation to awareness of airport-based screening measures, data were collected on previous awareness of fever screening and PCR testing for detected febrile passengers at airports in Okinawa and on the effect of increased pre-travel health awareness (Table 2). Previous awareness of fever screening at airports in Okinawa was 88.5% (2058/2326) among all participants, and its previous awareness among Okinawa residents (92.3%) (1390/1506) was significantly higher than among residents outside Okinawa (81.5%) (668/820) (*P* <0.001). A highly positive response to increased pre-travel health awareness was indicated by 94.1% (95% confidence interval [CI]: 93.0-95.1) (1925/2046) of respondents, regardless of residence. These high proportions suggest strong penetration of airport screening messages and widespread behavioral response among travelers. A similar tendency was found in the questionnaire on PCR testing for febrile passengers. Previous awareness of PCR testing for febrile passengers at airports in Okinawa was 77.1% (1785/2316) among all participants, and previous awareness among Okinawa residents (82.6%) (1238/1499) was significantly higher than among residents outside Okinawa (67.0%) (547/817) (*P* <0.001). A highly positive response (96.4% [95% CI: 95.4-97.2%]) (527/542) was shown in increasing pre-travel health awareness among the participants who were aware of its testing at the airport, regardless of residency. Together, these findings highlight a consistent pattern: awareness of screening measures corresponded with substantial increases in self-reported pre-travel health vigilance.

**Table 2:** Responses to key questionnaire items on previous awareness of airport health measures and self-reported increases in pre-travel health awareness among Naha Airport polymerase chain reaction Project participants.

		All participants		Okinawa		Outside of Okinawa		*P*-value
		Respondents	Answered ``yes'' n (%)	Respondents	Answered ``yes'' n (%)	Respondents	Answered ``yes'' n (%)	
	
Q1. Previous awareness of thermographic fever screening at airports in Okinawa.		2326	2058 (88.5)	1506	1390 (92.3)	820	668 (81.5)	<0.001^d^
aQ2. Increasing health awareness before the travel by thermographic fever screening		2046	1925 (94.1)	1385	1302 (94.0)	661	623 (94.3)	0.827
Q3. Previous awareness of PCR testing for febrile passengers at airports in Okinawa		2316	1785 (77.1)	1499	1238 (82.6)	817	547 (67.0)	<0.001^d^
bQ4. Increasing health awareness before the travel by PCR testing		1775	1711 (96.4)	1233	1184 (96.0)	542	527 (97.2)	0.209

a Q2 was asked only the person if Q1 was “yes”.

b Q4 was asked only the person if Q3 was “yes”. ^c^Bonferroni-adjusted *P*-values from a two-sample test for equality of proportions (no continuity correction); *P* <0.0125 regarded as statistically significant for this table.

d *P*<0.001.

Perceived safety of airport PCR testing was high, with ≥99% of respondents rating it non-negative (i.e., “safe”, “a little safe”, or “better than nothing”; Table S1); the most common response was “safe” (81.0%), and there were no residence-based differences. This uniformly positive perception suggests that voluntary airport testing may reduce psychological barriers to travel during epidemics.

Table 3 summarizes the results of an additional survey conducted in March 2021 to assess the passenger backgrounds related to COVID-19 infection and protection, and physical conditions with subjective symptoms. In March 2021, almost all respondents had never been vaccinated (98.8%) and had never had COVID-19 (98.8%), and 96.1% reported no subjective symptoms. Overall, 3.9% reported symptoms, most commonly a runny nose (64.4%), with fever reported by only 0.27% (Table 3).

**Table 3:** Additional survey in March 2021.

	n	(%)
Vaccination history (n = 1922, response proportion = 73.3%)		
Never	1898	(98.8)
Once	19	(1.0)
Twice	5	(0.3)
COVID-19 infection history (n = 1884, response proportion = 71.9%)		
Never	1861	(98.8)
Within 3 months	13	(0.7)
More than 3 months ago	10	(0.5)
Subjective symptoms (n = 1859, response proportion = 70.9%)		
Without	1786	(96.1)
With	73	(3.9)
^a^Type of symptoms in symptomatic persons (n = 73)		
Fever	5	(6.8)
Cough	14	(19.2)
Sputum	5	(6.8)
Runny nose	47	(64.4)
Sneezing	28	(38.4)
Breathlessness	0	(0.0)
Sore throat	4	(5.5)
Fatigue	5	(6.8)
Abnormal taste	0	(0.0)
Diarrhea	7	(9.6)
Travel history (n = 1839, response proportion = 70.1%)		
Within 1 month	10	(0.5)
More than 1 month ago, but within 1 year	18	(1.0)
More than 1 year	1811	(98.5)
^a^Source of knowing Naha Airport polymerase chain reaction Project (n = 1801, response proportion = 68.7%)		
TV/Newspaper	490	(27.2)
Internet News/ Social media	341	(18.9)
Homepage of Okinawa prefecture	205	(11.4)
Friends and relatives living in Okinawa	615	(34.1)
Friends and relatives outside Okinawa	72	(4.0)
Others	277	(15.4)
^a^Multiple answers were allowed.		

The proportion of participants reporting symptoms was examined by purpose of travel (Table 4). The proportion symptomatic for “Visiting friends and relatives”, “Business”, and “Sightseeing” was 4.63% (95% CI: 3.25-6.38) (35/756), 3.96% (95% CI: 2.23-6.44) (15/379), and 6.56% (95% CI: 2.87-12.51) (8/122), respectively. There was no statistically significant difference between these groups. Thus, travel purpose alone did not appear to drive symptom reporting in this population.

**Table 4:** Proportion symptomatic by purpose of travel (n = 1817).

	All participants		Subjective symptoms				*P*-value^a^	Proportion symptomatic
			Without		With			
	n	(%)	n	(%)	n	(%)		% (95% confidence interval)
Visiting friends and relatives	756	(41.6)	721	(41.3)	35	(48.6)	0.219	4.63 (3.25-6.38)
Business	379	(20.9)	364	(20.9)	15	(20.8)	0.996	3.96 (2.23-6.44)
Sightseeing	122	(6.7)	114	(6.5)	8	(11.1)	0.128	6.56 (2.87-12.51)
Other	560	(30.8)	546	(31.3)	14	(19.4)	0.033^b^	2.50 (1.37-4.16)
Total	1817	(100)	1745	(100)	72	(100)	-	3.96 (3.11-4.96)

Response proportion = 97.7%.

a Bonferroni-adjusted *P*-values from a two-sample test for equality of proportions (no continuity correction); *P* <0.0125 regarded as statistically significant for this table.

b *P* <0.05.

Next, we examined the proportion symptomatic by reason for testing (Table 5). The proportion symptomatic for “For family”, “Workplace mandates”, and “Personal concern” were 4.53% (95% CI: 3.28-6.08) (42/927), 1.60% (95% CI: 0.64-3.27) (7/438), and 4.58% (95% CI: 2.52-7.56) (14/306), respectively. The proportion symptomatic for those whose reason for testing was “Workplace mandates” was significantly lower than for those testing “For family” (4.54% vs 1.60%; *P* = 0.005), and the proportion with workplace-mandated testing was also lower among symptomatic than asymptomatic participants (10.0% vs 24.8%; *P* = 0.004) (Table 5). These descriptive differences suggested a behavioral pattern in which workplace-driven testers may have been more likely to defer travel when unwell.

**Table 5:** Proportion symptomatic by reason for testing (n = 1807).

	All participants		Proportion symptomatic				*P*-value^a^	Proportion symptomatic
			Without		With			
	n	(%)	n	(%)	n	(%)		% (95% confidence interval)
For family	927	(51.3)	885	(50.9)	42	(60.0)	0.147	4.54 (3.28-6.08)
Workplace mandates	438	(24.2)	431	(24.8)	7	(10.0)	0.004^b^	1.60 (0.64-3.27)
Personal concern	306	(16.9)	292	(16.8)	14	(20.0)	0.496	4.58 (2.52-7.56)
Other	136	(7.5)	129	(7.5)	7	(10.0)	0.430	5.15 (2.09-10.32)
Total	1807	(100)	1737	(100)	70	(100)	-	3.87(3.03-4.87)

Response rate = 97.2%.

a Bonferroni-adjusted *P*-values from a two-sample test for equality of proportions (no continuity correction); *P* <0.0125 regarded as statistically significant for this table.

b *P* <0.01.

Age distributions stratified by the reason for testing were broadly dispersed and did not account for the bimodal pattern; in contrast, stratification by the purpose of travel explained the two peaks (around the 20s and 50s) (Figure S2). A Sankey diagram further showed a strong alignment between visiting friends and relatives and family requests, and between business travel and workplace mandate, especially among non-Okinawa residents (Figure S3). Among participants with non-missing symptom data, 3.9% (73/1,859) reported symptoms. In univariate analyses, the proportion symptomatic was lower in ages 40-49 and 50-59 compared with under 30, while gender, vaccination history, and previous COVID-19 infection showed no clear associations; for the “Ever” categories in vaccination and previous infection, no symptomatic cases occurred (Supplementary Table S4). In the multivariable logistic model (Supplementary Table S3), workplace-mandated testing remained independently associated with lower odds of symptoms compared with family request (aOR 0.36, 95% CI 0.15-0.78), and the sensitivity Poisson model yielded consistent estimates (aRR 0.38, 95% CI 0.17-0.84; Supplementary Table S4). Residence was also associated with symptomatic status: in multivariable models, Okinawa residents had about half the odds of reporting symptoms compared with non-residents (aOR 0.50, 95% CI 0.30-0.82; aRR 0.52, 95% CI 0.33-0.82; Supplementary Tables S3-S4).

## Discussion

This study described self-reported changes in pre-travel health awareness associated with awareness of voluntary airport-based measures among domestic travelers. Among those aware of fever screening and PCR testing, more than 95% reported paying closer attention to their health before travel. Although this cross-sectional survey does not allow causal inference, the presence of symptoms in 3.9% of participants indicates that some individuals still chose to travel despite mild complaints, likely reflecting a combination of personal risk perception and perceived necessity of travel.

During the study period, negative certification of PCR tests before boarding was not mandatory for domestic travel by airplanes and ferries in Japan. In contrast, the Tokyo Metropolitan Government required negative PCR certification for passengers on certain ferries, suggesting that additional pre-boarding PCR testing may be a viable option [[18], [19], [20]], although some observational studies have reported weaknesses in pre-boarding testing [21,22]. In this context, our findings suggest that voluntary airport-based measures during this early pandemic phase may have functioned less as strict infection-detection tools and more as visible cues that shaped travelers’ pre-travel behavior and health awareness.

In the March 2021 OKA subset, the workplace-mandated testers consistently exhibited a lower proportion of symptomatic individuals than those testing for family reasons, and this association persisted after adjustment for demographics and COVID-19-related histories. A plausible explanation is that workplace policies may promote pre-travel self-screening or postponement when unwell, thereby reducing symptom reporting at the time of testing. The age gradient (lower proportion symptomatic in middle-aged groups vs under 30) aligns with differential health-seeking and reporting behaviors in this cohort, although the adjusted estimates for vaccination/previous-infection were not estimable due to zero events in “Ever” categories, reflecting the very low vaccination coverage and rare previous infection during early 2021. The lower symptom prevalence among Okinawa residents may reflect differences in travel patterns, thresholds for seeking testing, or local risk communication compared with non-residents, although our data do not allow us to distinguish these mechanisms. These findings are inherently observational and rely on self-reported symptoms; nonetheless, the direction and robustness across logistic and Poisson models strengthen the inference that the reason for testing is associated with symptom reporting in this setting (Supplementary Table S3).

Routine population-level data on the proportion symptomatic were not collected during the study period; therefore, we provide contextual but not directly comparable metrics based on official daily case counts from the MHLW dataset [17]. During February-March 2021, incidence in Okinawa and Japan overall remained modest, and the 3.9% of travelers reporting symptoms reflects symptoms—not confirmed infections—in a selected cohort of travelers, making direct comparison with population case incidence inappropriate.

Previous awareness of fever screening and PCR testing for febrile passengers at OKA reached approximately 80-90% among residents in Okinawa Prefecture, while previous awareness was significantly lower among residents outside Okinawa Prefecture by about 10-15%. These results indicate that while Okinawa Prefecture’s efforts to disseminate information to its residents were successful, there were challenges in communicating information to residents outside Okinawa. It is realistically difficult for the local government of the destination prefecture to alert the residents of the source prefecture, so efforts by the local government of the source prefecture and the Japanese government may be required.

The proportion of NAPP participants traveling for ``sightseeing'' was low (4.6%), despite an estimated 418 thousand inbound tourists to Okinawa Prefecture during the study period [23]. This likely reflects limited awareness of NAPP among tourists and the potential disincentive of being quarantined after a positive test, which would affect their companions. These findings highlight the need for targeted communication and facilitation of voluntary testing for tourists, tailored to travel purpose and age group.

The uniformly high perceived safety suggests that voluntary PCR screening may help reduce the psychological burden of traveling during a pandemic, particularly for unavoidable business or other essential trips. The low vaccination coverage among participants reflects the early stage of vaccine rollout in Japan at this time [24]. The overwhelmingly positive perception of safety also indicates that voluntary testing programs may serve not only epidemiological roles but also important reassurance functions during public health emergencies.

Our study is not without limitations. First, our questionnaire survey was restricted to participants in the NAPP and did not cover all arriving passengers. During the study period, 4,545 of 5,308 NAPP participants (85.6%) completed the questionnaire, indicating high coverage within the program; however, because NAPP itself included only about 0.87% of all arriving passengers [5], our findings may not be fully generalizable to all domestic travelers to Okinawa. Second, the external validity of the results is an issue because the survey period was only 2 months immediately after the start of the NAPP project, and there is a participant selection bias considering the small fraction of tourists among the total NAPP respondents. However, while these may have affected the rate of pre-travel health awareness, they do not undermine the perceived association between airport-based screening and travelers’ health awareness. Third, because test results could not be linked to the de-identified survey data, we cannot report respondent-level positivity; our findings pertain to perceptions and self-reported symptoms rather than infection prevalence. These findings will provide important evidence for airport screening measures in future pandemics.

## Conclusion

Participants who were aware of airport-based health measures such as fever screening and PCR testing reported greater attention to their own health before travel, suggesting a psychological influence of visible screening measures on health-related behavior. Among voluntary airport PCR testers, workplace-mandated testing was associated with fewer self-reported symptoms than family-driven testing, indicating that how testing is initiated may shape travelers’ pre-travel self-management. Although based on cross-sectional, self-reported data and not suited for causal inference, these findings suggest that voluntary pre-boarding testing may function less as a primary infection-control tool than as a practical behavioral nudge to support health-conscious travel decisions.

## Funding

This work was supported by the Japan Society for the Promotion of Science (JSPS) (Grant 20H03940, 20KK0367 to KM, 21K10416 to YS, 21K17250 to RM); and by the Japan Science and Technology Agency (JST) (Grant JPMJSC21U4, JPMJFR215G to KM, JPMJCR20H1 to RO).

## Ethical approval

This study was conducted to support an ongoing public health response. Ethical approval and the requirement for informed consent for this study were waived by the Okinawa Prefecture Department of Culture, Tourism and Sports.

## Author contribution

Masahiro Kozuka: Data collection, formal analysis, writing – original draft; Yusuke Shimakawa: Writing – review & editing; Gerardo Chowell: Writing – review & editing; Tetsuharu Nagamoto: Writing – review & editing; Ryota Matsuyama: Writing – review & editing; Ryosuke Omori: Writing – review & editing; Yining S. Xu: Writing – review & editing; Yoshiro Shimoji: Data collection coordination (Okinawa Prefecture); Kaoru Ogawa: Formal analysis (revised analyses); Yoshihiro Takayama: Study supervision (Okinawa Prefecture), data access approval; Kenji Mizumoto: Conceptualization, study design, formal analyses, writing – review & editing, supervision.

## Data availability

The data that support the findings of this study are available from the corresponding author and Okinawa Prefecture Government, but restrictions apply to the availability of these data, which were used under license for the current study, and so are not publicly available. Data are, however, available from the authors upon reasonable request and with permission of the Okinawa Prefecture Government.

## Declaration of generative AI and AI-assisted technologies in the writing process

During the preparation of this work, the author(s) used ChatGPT to improve language and readability. After using this tool/service, the author(s) reviewed and edited the content as needed and take(s) full responsibility for the content of the publication.

## Declaration of competing interest

The authors have no competing interests to declare.

## Appendix Supplementary materials

Image, application 1

## References

[1] Gostic K.M., Kucharski A.J., Lloyd-Smith J.O. (2015). Effectiveness of traveller screening for emerging pathogens is shaped by epidemiology and natural history of infection. eLife.

[2] Centers for Disease Control and Prevention (US) (2022). Requirement for proof of negative COVID-19 test result or recovery from COVID-19 for all airline passengers arriving into the United States. https://stacks.cdc.gov/view/cdc/113812.

[3] Ministry of Health, Labour and Welfare (2020). Response at the quarantine station. https://www.forth.go.jp/news/20200129_00004.html.

[4] Shaum A., Figueroa A., Lee D., Ertl A., Rothney E., Borntrager D. (2022). Cross-sectional survey of SARS-CoV-2 testing at US airports and one health department's proactive management of travelers. Trop Dis Travel Med Vaccin.

[5] Takayama Y., Xu Y.S., Shimakawa Y., Chowell G., Kozuka M., Omori R. (2024). Assessment of fever screening at airports in detecting domestic passengers infected with SARS-CoV-2, 2020–2022, Okinawa Prefecture, Japan. BMC Infect Dis.

[6] Normile D. (2020). Airport screening is largely futile, research shows. Science.

[7] Dollard P., Griffin I., Berro A., Cohen N.J., Singler K., Haber Y. (2020). Risk assessment and management of COVID-19 among travelers arriving at designated U.S. airports, January 17–September 13, 2020. MMWR Morb Mortal Wkly Rep.

[8] Quilty B.J., Clifford S. (2020). CMMID nCoV working group2, Flasche S, Eggo RM. Effectiveness of airport screening at detecting travellers infected with novel coronavirus (2019–nCoV). Euro Surveill.

[9] Nishiura H., Kamiya K. (2011). Fever screening during the influenza (H1N1-2009) pandemic at Narita International Airport, Japan. BMC Infect Dis.

[10] Gunaratnam P.J., Tobin S., Seale H., Marich A., McAnulty J. (2014). Airport arrivals screening during pandemic (H1N1) 2009 influenza in New South Wales, Australia. Med J Aust.

[11] Sakaguchi H., Tsunoda M., Wada K., Ohta H., Kawashima M., Yoshino Y., Aizawa Y. (2012). Assessment of border control measures and community containment measures used in Japan during the early stages of Pandemic (H1N1) 2009. PLoS One.

[12] Japan Airlines. Do I need a PCR test or vaccination certificate before boarding?, https://faq-en.jal.co.jp/app/answers/detail/a_id/27188/~/do-i-need-a-pcr-test-or-vaccination-certificate-before-boarding%3F; n.d. [accessed 03 June 2024].

[13] All Nippon Airways. Domestic flights: are negative certification for the COVID-19 and vaccination certificates required for boarding? Frequently Asked Questions, https://ana-support.my.site.com/jajp/s/article/answers000022235ja; n.d. [accessed 03 June 2024].

[14] Okinawa Prefecture (2021). Meeting of the Headquarters for Countermeasures to Combat COVID-19 held to discuss the NAPP. https://www.pref.okinawa.jp/kensei/kencho/1001519/1001649/1002254/1002256.html.

[15] Okinawa Prefecture (2021). Okinawa declaration of emergency: the, 75th Meeting of the Okinawa Prefectural Task Force on COVID-19. https://www.pref.okinawa.lg.jp/_res/projects/default_project/_page_/001/023/403/vol75siryou.pdf.

[16] Okinawa Prefecture-19 (2021). Fifth Meeting of Organizations concerned with Economic Countermeasures for the Impact of the COVID. https://www.pref.okinawa.jp/_res/projects/default_project/_page_/001/010/299/5th_gijigaiyou.pdf.

[17] Ministry of Health (2023). Labour and Welfare. Trends in infected patients, 2023. Data from the Data - Information on COVID-19 Infections. https://covid19.mhlw.go.jp/.

[18] Ogasawara Village, Tokyo Metropolitan Government [notice] (2021). Please cooperate with the PCR test before boarding the Ogasawara Maru, 2021 Nov 11. https://www.vill.ogasawara.tokyo.jp/panel-top_topics/soumuka/%EF%BD%9E%E3%81%8A%E3%81%8C%E3%81%95%E3%82%8F%E3%82%89%E4%B8%B8%E4%B9%97%E8%88%B9%E5%89%8D%E3%81%AEpcr%E6%A4%9C%E6%9F%BB%E3%81%AB%E3%81%94%E5%8D%94%E5%8A%9B%E3%81%8F%E3%81%A0%E3%81%95%E3%81%84.html/29423/.

[19] Ogasawara Village, Tokyo Metropolitan Government [notice] (2023). PCR testing for Ogasawara Maru passengers will be terminated, 2023 Apr 10. https://www.vill.ogasawara.tokyo.jp/panel-top_topics/soumuka/%E3%81%8A%E3%81%8C%E3%81%95%E3%82%8F%E3%82%89%E4%B8%B8%E4%B9%97%E8%88%B9%E5%AE%A2%E3%82%92%E5%AF%BE%E8%B1%A1%E3%81%A8%E3%81%97%E3%81%9F%E3%80%8Cpcr%E6%A4%9C%E6%9F%BB%E3%80%8D%E3%82%92%E7%B5%82.html/36417/.

[20] Terada-Hirashima J., Sugiura W., Shimizu Y., Tanaka Y., Uemura Y., Ishikane M. (2022). Investigation of the use of PCR testing prior to ship boarding to prevent the spread of SARS-CoV-2 from urban areas to less-populated remote islands. Glob Health Med.

[21] Molero-Salinas A., Rico-Luna C., Losada C., Buenestado-Serrano S., de la Cueva García V.M., Egido J. (2022). High SARS-CoV-2 viral load in travellers arriving in Spain with a negative COVID-19 test prior to departure. J Travel Med.

[22] Garcia-Bereguiain M.A., Perez F., Parra-Vera H., Bruno A., Freire-Paspuel B., Morales-Jadan D., Castro-Rodriguez B. (2022). High SARS-CoV-2 viral load in travellers arriving in Spain with a negative COVID-19 test prior to departure: Ecuador as a model for COVID-19 testing quality in Latin America. J Travel Med.

[23] Department of Culture, Tourism and Sports. Okinawa Prefecture (2022). Statistics on visitors to Okinawa in 2021. https://www.pref.okinawa.jp/_res/projects/default_project/_page_/001/026/300/r3-gaikyou.pdf.

[24] National Institute of Infectious Diseases (2021). Japan. About the COVID-19 vaccine (as of August 5, 2021). https://www.niid.go.jp/niid/ja/diseases/ka/corona-virus/2019-ncov/2484-idsc/10569-covid19-53.html.

