# Supplementary material for: Pre-travel health awareness and perceptions of voluntary airport PCR testing during COVID-19: A cross-sectional study in Okinawa, Japan
Source: IJID Reg. 2025 Nov 30;18:100817. doi: 10.1016/j.ijregi.2025.100817 (PMC12774711; doi:10.1016/j.ijregi.2025.100817)
Supplement: Supplementary file 1 [file mmc1.docx]

**Supplementary information**

Pre-travel health awareness and perceptions of voluntary airport PCR testing during COVID-19: A cross-sectional study in Okinawa, Japan

**Supplementary information:**

The electronic supplementary material is below the table titles and figures.

1. **Supplementary Results**
2. **Supplementary Tables S1-S4**
3. **Supplementary Figures S1-S4**

**Supplementary Results:**

During February–March 2021, Okinawa reported 2.19 daily cases per 100,000 (7-day moving average; range 0.89–5.56), while Japan overall reported 1.19 (range 0.78–2.65), based on October 1, 2021 population estimates. As separate program-level context, official reports on the Naha Airport PCR testing program documented 4,869 testers with 7 positives as of 28 March 2021 (0.14%) and 5,824 testers with 14 positives as of 4 April 2021 (0.24%) [1,2]; these aggregates include both residents and non-residents, cover slightly different time windows than our survey, and were not linkable to our de-identified dataset. For scale only, the 3.9% of travelers reporting symptom corresponds to 3,900 per 100,000 travelers, but this reflects symptoms (not confirmed infections) in a selected cohort and is therefore not commensurable with population case incidence per 100,000 residents.

1. Okinawa Prefecture. COVID-19 outbreak situation report, the 86th task force meeting (FY 2021), Document 10, status of NAPP, TACO and RICCA [Internet]. 2021 Mar 29. [cited 2024 Jul 3]. Available from: https://www.pref.okinawa.jp/_res/projects/default_project/_page_/001/023/403/siryo2_0329.pdf. (in Japanese).
2. Okinawa Prefecture. COVID-19 outbreak situation report, the 87th task force meeting, Document 10, status of NAPP, TACO and RICCA [Internet]. 2021 Apr 5. [cited 2024 Jul 3]. Available from: https://www.pref.okinawa.jp/_res/projects/default_project/_page_/001/023/403/shiryo2.pdf. (in Japanese).

Table S1. The questionnaire for safety feelings for PCR testing at airports (n = 2327)

|  | n (%) | | | | | | p-value |
| --- | --- | --- | --- | --- | --- | --- | --- |
|  | All participants | | Residence | | | |  |
|  |  |  | Okinawa | | Outside of Okinawa | |  |
| Safe | 1884 | (81.0) | 1222 | (80.9) | 662 | (81.0) | 0.953 |
| A little safe | 247 | (10.6) | 157 | (10.4) | 90 | (11.0) | 0.644 |
| Better than nothing | 184 | (7.9) | 123 | (8.1) | 61 | (7.5) | 0.562 |
| Not effective | 7 | (0.3) | 4 | (0.3) | 3 | (0.4) | 0.667 |
| Useless | 5 | (0.2) | 4 | (0.3) | 1 | (0.1) | 0.479 |
| Total | 2327 | (100) | 1510 | (100) | 817 | (100) | - |
| *Bonferroni-adjusted p-values P<0.0125 (significant) using a 2-sample test for equality of proportions without continuity correction | | | | | | | |
| p-values symbols: *** p<0.001; ** p<0.01; * p<0.05; . p<0.1 | | | | | | | |

**Table S2. Original questionnaire survey (in Japanese)**

| 1 | 渡航目的を選択して下さい。 |
| --- | --- |
| 2 | 検査を受ける理由を選択して下さい。 |
| 3 | 出発地で受けない理由を選択してください。 |
| 4 | 沖縄県が空港でサーモグラフィー発熱感知検査を行っていることを知っていましたか。 |
| 5 | 問4が「はい」の場合、サーモグラフィー発熱感知検査があることで旅行前の体調管理をより徹底するよう意識しましたか。 |
| 6 | 沖縄県が空港等で発熱がある方にPCR検査を行っていることを知っていましたか。 |
| 7 | 問6が「はい」の場合、水際で発熱がある方にPCR検査を行っていることで旅行前の体調管理をより徹底するよう意識しましたか。 |
| 8 | 沖縄県が空港で発熱者や希望者にPCR検査を実施する体制を整備していることについてどう思いますか。 |

Table S3. Multivariable logistic regression of symptomatic status among passengers arriving at OKA in March 2021 (n = 1,859)

| Variable | Category | Adjusted odds ratio (aOR) | 95% CI | p-value |
| --- | --- | --- | --- | --- |
| Reason for testing | For family | 1.00 (reference) | – | – |
|  | Workplace | 0.36 | 0.15–0.78 | 0.02 * |
|  | Personal concern | 1.22 | 0.63–2.25 | 0.54 |
|  | Other | 1.11 | 0.44–2.43 | 0.80 |
| Age group (years) | Under 30 | 1.00 (reference) | – | – |
|  | 30–39 | 0.95 | 0.44–1.92 | 0.90 |
|  | 40–49 | 0.43 | 0.17–0.94 | <0.05 * |
|  | 50–59 | 0.47 | 0.20–0.98 | 0.06 . |
|  | ≥60 | 0.88 | 0.40–1.77 | 0.74 |
| Gender | Female | 1.00 (reference) | – | – |
|  | Male | 1.04 | 0.63–1.70 | 0.88 |
| Vaccination history | Never | 1.00 (reference) | – | – |
|  | Ever | – | N.E. | N.E. |
| COVID-19 infection | Never | 1.00 (reference) | – | – |
|  | Ever | – | N.E. | N.E. |
| Residence | Outside Okinawa | 1.00 (reference) | – | – |
|  | Okinawa | 0.50 | 0.30–0.82 | <0.01 ** |

OR, odds ratio; CI, confidence interval; N.E. = not estimable.

p-values symbols: *** p<0.001; ** p<0.01; * p<0.05; . p<0.1

Symptomatic status was defined as having any self-reported symptom (“with” in the questionnaire).

Odds ratios were obtained from a multivariable logistic regression including reason for testing, age group, gender, vaccination history, history of COVID-19 infection, and residence as covariates. Reference categories were: For family (purpose of test), Under 30 (age group), Female (gender), Never (vaccination history), Never (COVID-19 infection history), and Outside Okinawa (residence).

For vaccination history and prior COVID-19 infection, no symptomatic cases occurred in the “Ever” category; therefore, adjusted odds ratios and 95% CIs could not be estimated and are shown as N.E. in the table.

Table S4. Univariate and multivariable associations between participant characteristics and symptomatic status (Poisson regression with robust variance, n = 1,859)

| Variable | Category | n | Symptomatic, n (%) | Univariate RR | 95% CI | p-value | Multivariable adjusted RR | 95% CI | p-value |
| --- | --- | --- | --- | --- | --- | --- | --- | --- | --- |
| Age group (years) | Under 30 | 697 | 36 (5.2) | 1.00 (ref) | – | – | 1.00 (ref) | – | – |
|  | 30–39 | 239 | 11 (4.6) | 0.89 | 0.46–1.72 | 0.73 | 0.96 | 0.47–1.94 | 0.90 |
|  | 40–49 | 352 | 8 (2.3) | 0.44 | 0.21–0.94 | 0.03 * | 0.45 | 0.20–1.02 | 0.06 . |
|  | 50–59 | 364 | 8 (2.2) | 0.43 | 0.20–0.91 | 0.03 * | 0.48 | 0.23–1.03 | 0.06 . |
|  | ≥60 | 207 | 10 (4.8) | 0.94 | 0.47–1.85 | 0.85 | 0.89 | 0.45–1.75 | 0.73 |
| Gender | Female | 904 | 38 (4.2) | 1.00 (ref) | – | – | 1.00 (ref) | – | – |
|  | Male | 955 | 35 (3.7) | 0.87 | 0.56–1.37 | 0.55 | 1.04 | 0.65–1.66 | 0.88 |
| Vaccination history | Never | 1833 | 73 (4.0) | 1.00 (ref) | – | – | 1.00 (ref) | – | – |
|  | Ever | 24 | 0 (0.0) | – | N.E. | N.E. | – | N.E. | N.E. |
| COVID-19 infection | Never | 1804 | 72 (4.0) | 1.00 (ref) | – | – | 1.00 (ref) | – | – |
|  | Ever | 22 | 0 (0.0) | – | N.E. | N.E. | – | N.E. | N.E. |
| Reason for testing | For family | 927 | 42 (4.5) | 1.00 (ref) | – | – | 1.00 (ref) | – | – |
|  | Workplace | 438 | 7 (1.6) | 0.35 | 0.16–0.78 | <0.01 ** | 0.38 | 0.17–0.84 | 0.02 * |
|  | Personal concern | 306 | 14 (4.6) | 1.01 | 0.56–1.82 | 0.97 | 1.21 | 0.66–2.20 | 0.54 |
|  | Other | 136 | 7 (5.1) | 1.14 | 0.52–2.48 | 0.75 | 1.10 | 0.51–2.41 | 0.80 |
| Residence | Outside | 610 | 35 (5.7) | 1.00 (ref) | – | – | 1.00 (ref) | – | – |
|  | Okinawa | 1249 | 38 (3.0) | 0.53 | 0.34–0.83 | <0.01 ** | 0.52 | 0.33–0.82 | <0.01 ** |

RR, risk ratio; CI, confidence interval; N.E. = not estimable.

p-values symbols: *** p<0.001; ** p<0.01; * p<0.05; . p<0.1.

RRs and 95% CIs were estimated from Poisson regression models with robust variance. Univariate RRs were obtained from models including each variable separately; adjusted RRs were obtained from a multivariable model including age group, gender, vaccination history, prior COVID-19 infection, residence, and purpose of test.

Reference categories were: Under 30 (age group), Female (gender), Never (vaccination history), Never (COVID-19 infection history), Outside Okinawa (residence), and For family (purpose of test).

No symptomatic cases occurred in the “Ever” categories for vaccination history or prior COVID-19 infection; thus, the corresponding RRs and 95% CIs could not be estimated and are shown as N.E. in the table.

# Supplementary Figures:

## Figure S1 – Histogram of the age distribution for arriving passengers in Naha Airport who participated in NAPP.





All the arriving passengers at Naha Airport who participated in NAPP were distributed by the age (A) All arriving passengers. (B) Arriving passengers who are residents of Okinawa Prefecture. (C) Arriving passengers who are not residents of Okinawa Prefecture

## Figure S2 – Violin and box plots of purposes of travel and reasons for testing by age.





(A)(B)(C): Purposes of travel. (D)(E)(F): Reason for testing.

All arriving passengers who participated in NAPP at Naha Airport were grouped by age: (A) all passengers; (B) Okinawa residents; (C) non-residents.

## Figure S3 – Sankey diagram for the purposes of travel and reason for testing.


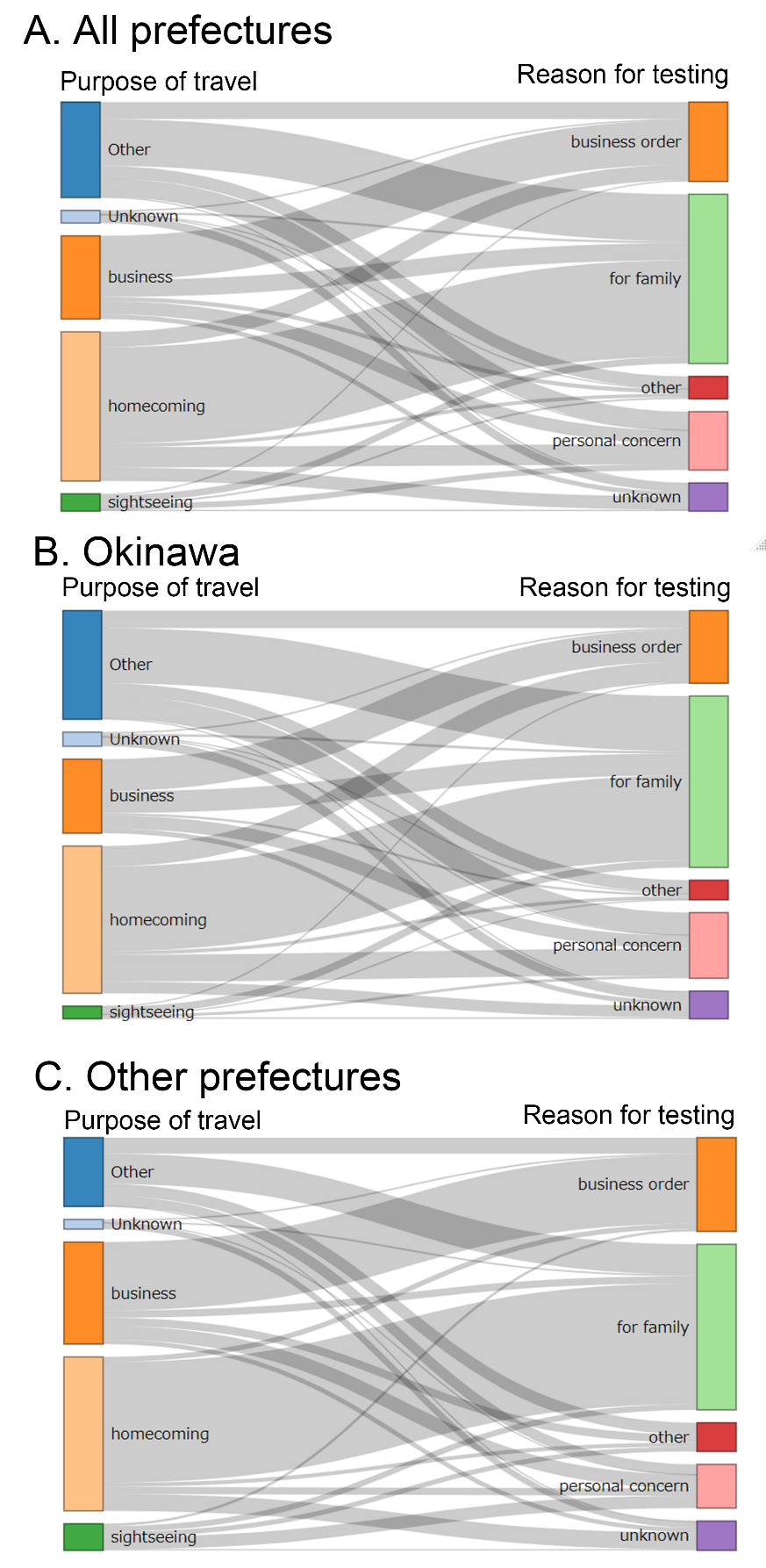


All arriving passengers at Naha Airport who participated in the NAPP were divided as follows. (A) All arriving passengers. (B) Arriving passengers who are residents of Okinawa Prefecture. (C) Arriving passengers who are not residents of Okinawa Prefecture

**Figure S4. Temporal distribution of COVID-19 cases stratified by age group in Japan and Okinawa Prefecture, September 2020–June 2021.**


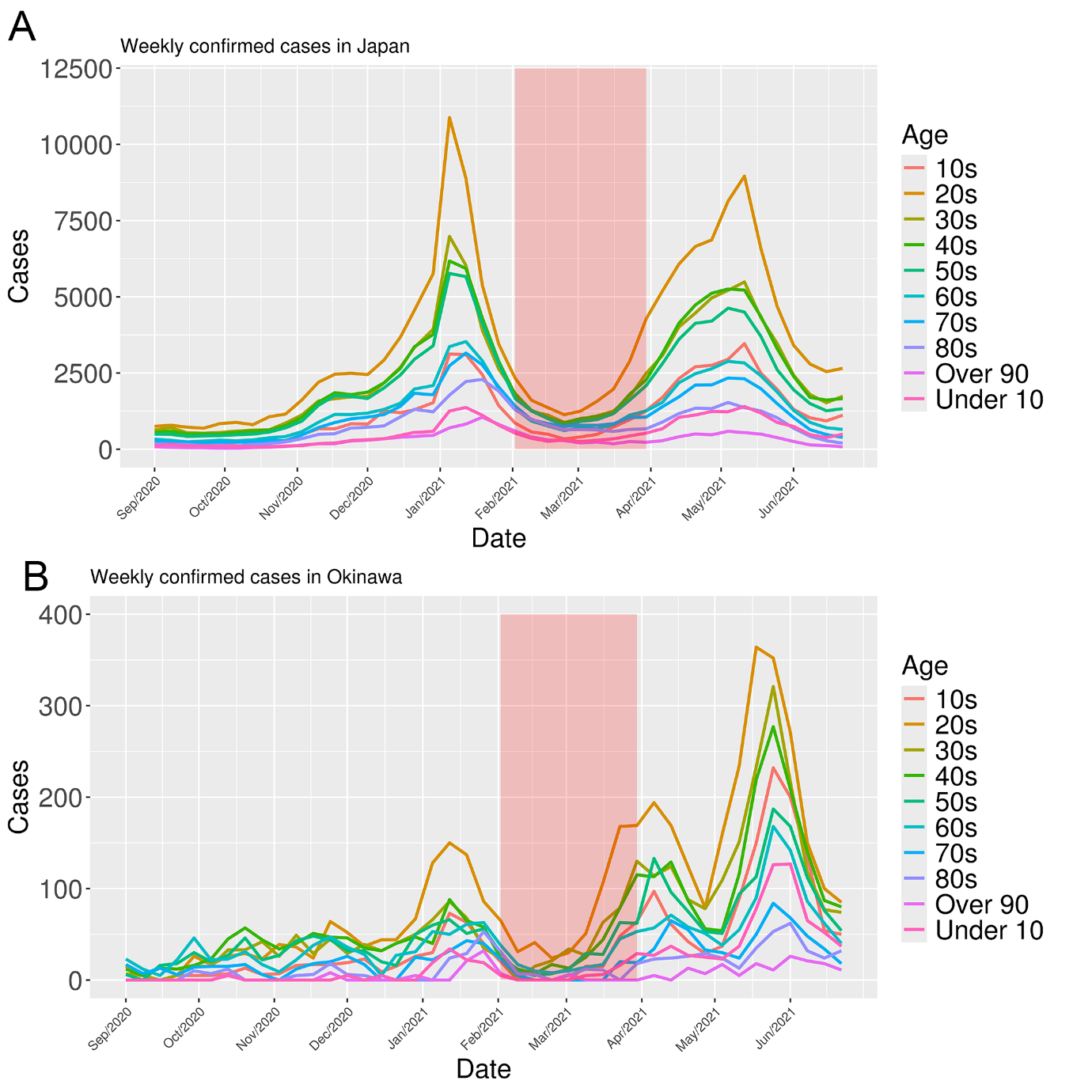


(A) Weekly confirmed cases in Japan, (B) Weekly confirmed cases in Okinawa Prefecture
